# Supplementary material for: The impact of screen time and social media on youth self-harm behaviour and suicide: A protocol for a systematic reviews
Source: PLoS One. 2024 Dec 2;19(12):e0314621. doi: 10.1371/journal.pone.0314621 (PMC11611183; doi:10.1371/journal.pone.0314621)
Supplement: S2 Appendix — (DOCX) [file pone.0314621.s002.docx]

**S2 Appendix: Search Strategy**

| **Database** | **Date** | **Search number** | **Query** | **Results** |
| --- | --- | --- | --- | --- |
| CINAHL |  | S1 | screentime OR “screen time” OR smartphone OR “smart phone” OR “social media” OR internet OR gaming OR texting OR television OR “video games” OR messaging OR “smart device” OR phone OR telephone OR computer OR laptop OR tablet OR “Chat room” or online or “social network” OR blog OR sext* OR “instant messag*” or “text messag*” OR cyber* OR forum OR YouTube OR reddit OR twitter OR Snapchat OR Instagram OR Facebook OR telegram OR WeChat OR WhatsApp OR TikTok OR discord OR Twitch OR myspace OR Kuaishou OR Qzone OR “Seina Weibo” or QQ OR Threads OR Mastadon OR bluesky OR Tumblr OR Rumble OR Quora |  |
|  |  | S2 | Child OR adolescent OR infant OR youth |  |
|  |  | S3 | “Self-harm” OR “self harm” OR “self-cut*” OR “self-mutilat*” OR “self-injur*” OR suicide OR suicidal |  |
|  |  | S4 | (MH "Injuries, Self-Inflicted") |  |
|  |  | S5 | (MH "Suicide+") OR (MH "Suicide, Attempted") OR (MH "Suicidal Ideation") |  |
|  |  | S6 | S3 OR S4 OR S5 |  |
|  |  | S7 | S1 AND S2 AND S6 |  |
| PubMed |  | #1 | “screentime"[Other Term] OR “screen time"[Other Term] "smartphone"[Other Term] OR "smart phone"[Other Term] OR "social media"[Other Term] OR "internet"[Other Term] OR "gaming"[Other Term] OR "texting"[Other Term] OR "television"[Other Term] OR "video games"[Other Term] OR "messaging"[Other Term] OR "smart device"[Other Term] OR "phone"[Other Term] OR "telephone"[Other Term] OR "computer"[Other Term] OR "laptop"[Other Term] OR "tablet"[Other Term] OR "Chat room"[Other Term] OR "online"[Other Term] OR "social network"[Other Term] OR "blog"[Other Term] OR "sext*"[Other Term] OR "instant messag*"[Other Term] OR "text messag*"[Other Term] OR "cyber*"[Other Term] OR "forum"[Other Term] OR "YouTube"[Other Term] OR "reddit"[Other Term] OR "twitter"[Other Term] OR "Snapchat"[Other Term] OR "Instagram"[Other Term] OR "Facebook"[Other Term] OR "telegram"[Other Term] OR "WeChat"[Other Term] OR "WhatsApp"[Other Term] OR "TikTok"[Other Term] OR "discord"[Other Term] OR "Twitch"[Other Term] OR "myspace"[Other Term] OR "Qzone"[Other Term] OR "QQ"[Other Term] OR "Threads"[Other Term] OR "bluesky"[Other Term] OR "Tumblr"[Other Term] OR "Rumble"[Other Term] OR "Quora"[Other Term] |  |
|  |  | #2 | screentime"[Title/Abstract] OR "screen time"[Title/Abstract] OR "smartphone"[Title/Abstract] OR "smart phone"[Title/Abstract] OR "social media"[Title/Abstract] OR "internet"[Title/Abstract] OR "gaming"[Title/Abstract] OR "texting"[Title/Abstract] OR "television"[Title/Abstract] OR "video games"[Title/Abstract] OR "messaging"[Title/Abstract] OR "smart device"[Title/Abstract] OR "phone"[Title/Abstract] OR "telephone"[Title/Abstract] OR "computer"[Title/Abstract] OR "laptop"[Title/Abstract] OR "tablet"[Title/Abstract] OR "Chat room"[Title/Abstract] OR "online"[Title/Abstract] OR "social network"[Title/Abstract] OR "blog"[Title/Abstract] OR "sext*"[Title/Abstract] OR "instant messag*"[Title/Abstract] OR "text messag*"[Title/Abstract] OR "cyber*"[Title/Abstract] OR "forum"[Title/Abstract] OR "YouTube"[Title/Abstract] OR "reddit"[Title/Abstract] OR "twitter"[Title/Abstract] OR "Snapchat"[Title/Abstract] OR "Instagram"[Title/Abstract] OR "Facebook"[Title/Abstract] OR "telegram"[Title/Abstract] OR "WeChat"[Title/Abstract] OR "WhatsApp"[Title/Abstract] OR "TikTok"[Title/Abstract] OR "discord"[Title/Abstract] OR "Twitch"[Title/Abstract] OR "myspace"[Title/Abstract] OR "Kuaishou"[Title/Abstract] OR "Qzone"[Title/Abstract] OR "QQ"[Title/Abstract] OR "Threads"[Title/Abstract] OR "bluesky"[Title/Abstract] OR "Tumblr"[Title/Abstract] OR "Rumble"[Title/Abstract] OR "Quora"[Title/Abstract] |  |
|  |  | #3 | #1 OR #2 |  |
|  |  | #4 | "Self-harm"[Other Term] OR "Self-harm"[Other Term] OR "self cut*"[Other Term] OR "self mutilat*"[Other Term] OR "self injur*"[Other Term] OR "suicide"[Other Term] OR "suicidal"[Other Term] |  |
|  |  | #5 | "Self-harm"[Title/Abstract] OR "Self-harm"[Title/Abstract] OR "self cut*"[Title/Abstract] OR "self mutilat*"[Title/Abstract] OR "self injur*"[Title/Abstract] OR "suicide"[Title/Abstract] OR "suicidal"[Title/Abstract] |  |
|  |  | #6 | "suicide, attempted"[MeSH Terms] OR "self injurious behavior"[MeSH Terms] OR "self injurious behavior"[MeSH Terms] |  |
|  |  | #7 | #4 OR #5 OR #6 |  |
|  |  | #8 | "Child"[Other Term] OR "adolescent"[Other Term] OR "infant"[Other Term] OR "youth"[Other Term] |  |
|  |  | #9 | "Child"[Title/Abstract] OR "adolescent"[Title/Abstract] OR "infant"[Title/Abstract] OR "youth"[Title/Abstract] |  |
|  |  | #10 | #8 OR #9 |  |
|  |  | #11 | #3 AND #7 AND #10 |  |
| Embase |  | #1 | screentime:ti,ab,kw OR 'screen time':ti,ab,kw OR smartphone:ti,ab,kw OR 'smart phone':ti,ab,kw OR 'social media':ti,ab,kw OR internet:ti,ab,kw OR gaming:ti,ab,kw OR texting:ti,ab,kw OR television:ti,ab,kw OR 'video games':ti,ab,kw OR messaging:ti,ab,kw OR 'smart device':ti,ab,kw OR phone:ti,ab,kw OR telephone:ti,ab,kw OR computer:ti,ab,kw OR laptop:ti,ab,kw OR tablet:ti,ab,kw OR 'chat room':ti,ab,kw OR online:ti,ab,kw OR 'social network':ti,ab,kw OR blog:ti,ab,kw OR sext*:ti,ab,kw OR 'instant messag*':ti,ab,kw OR 'text messag*':ti,ab,kw OR cyber*:ti,ab,kw OR forum:ti,ab,kw OR youtube:ti,ab,kw OR reddit:ti,ab,kw OR twitter:ti,ab,kw OR snapchat:ti,ab,kw OR instagram:ti,ab,kw OR facebook:ti,ab,kw OR telegram:ti,ab,kw OR wechat:ti,ab,kw OR whatsapp:ti,ab,kw OR tiktok:ti,ab,kw OR discord:ti,ab,kw OR twitch:ti,ab,kw OR myspace:ti,ab,kw OR kuaishou:ti,ab,kw OR qzone:ti,ab,kw OR 'seina weibo':ti,ab,kw OR qq:ti,ab,kw OR threads:ti,ab,kw OR mastadon:ti,ab,kw OR bluesky:ti,ab,kw OR tumblr:ti,ab,kw OR rumble:ti,ab,kw OR quora:ti,ab,kw |  |
|  |  | #2 | 'self-harm':ti,ab,kw OR 'self harm':ti,ab,kw OR 'self-cut*':ti,ab,kw OR 'self-mutilat*':ti,ab,kw OR 'self-injur*':ti,ab,kw OR suicide:ti,ab,kw OR suicidal:ti,ab,kw |  |
|  |  | #3 | 'suicidal behavior'/exp |  |
|  |  | #4 | 'suicide'/exp |  |
|  |  | #5 | 'automutilation'/exp |  |
|  |  | #6 | 'suicidal ideation'/exp |  |
|  |  | #7 | #2 OR #3 OR #4 OR #5 OR #6 |  |
|  |  | #8 | child:ti,ab,kw OR adolescent:ti,ab,kw OR infant:ti,ab,kw OR youth:ti,ab,kw |  |
|  |  | #9 | #1 AND #2 AND #3 |  |
| PsycArticles |  | S1 | screentime OR “screen time” OR smartphone OR “smart phone” OR “social media” OR internet OR gaming OR texting OR television OR “video games” OR messaging OR “smart device” OR phone OR telephone OR computer OR laptop OR tablet OR “Chat room” or online or “social network” OR blog OR sext* OR “instant messag*” or “text messag*” OR cyber* OR forum OR YouTube OR reddit OR twitter OR Snapchat OR Instagram OR Facebook OR telegram OR WeChat OR WhatsApp OR TikTok OR discord OR Twitch OR myspace OR Kuaishou OR Qzone OR “Seina Weibo” or QQ OR Threads OR Mastadon OR bluesky OR Tumblr OR Rumble OR Quora |  |
|  |  | S2 | “Self-harm” OR “self harm” OR “self-cut*” OR “self-mutilat*” OR “self-injur*” OR suicide OR suicidal |  |
|  |  | S3 | MA suicide OR MA suicide attempt OR MA suicide ideation OR MA ( self harm or self injury or self mutilation ) |  |
|  |  | S4 | S2 OR S3 |  |
|  |  | S5 | Child OR adolescent OR infant OR youth |  |
|  |  | S6 | S1 AND S4 AND S5 |  |
| PsycINFO |  | S1 | screentime OR “screen time” OR smartphone OR “smart phone” OR “social media” OR internet OR gaming OR texting OR television OR “video games” OR messaging OR “smart device” OR phone OR telephone OR computer OR laptop OR tablet OR “Chat room” or online or “social network” OR blog OR sext* OR “instant messag*” or “text messag*” OR cyber* OR forum OR YouTube OR reddit OR twitter OR Snapchat OR Instagram OR Facebook OR telegram OR WeChat OR WhatsApp OR TikTok OR discord OR Twitch OR myspace OR Kuaishou OR Qzone OR “Seina Weibo” or QQ OR Threads OR Mastadon OR bluesky OR Tumblr OR Rumble OR Quora |  |
|  |  | S2 | “Self-harm” OR “self harm” OR “self-cut*” OR “self-mutilat*” OR “self-injur*” OR suicide OR suicidal |  |
|  |  | S3 | MA ( self harm or self injury or self mutilation ) OR MA ( suicide or suicidal ideation or suicidality or suicide attempts ) |  |
|  |  | S4 | S2 OR S3 |  |
|  |  | S5 | Child OR adolescent OR infant OR youth |  |
|  |  | S6 | S1 AND S4 AND S5 |  |
| Scopus |  | S1 | TITLE-ABS-KEY ( screentime OR “screen time” OR smartphone OR "smart phone" OR "social media" OR internet OR gaming OR texting OR television OR "video games" OR messaging OR "smart device" OR phone OR telephone OR computer OR laptop OR tablet OR "Chat room" OR online OR "social network" OR blog OR sext* OR "instant messag*" OR "text messag*" OR cyber* OR forum OR youtube OR reddit OR twitter OR snapchat OR instagram OR facebook OR telegram OR wechat OR whatsapp OR tiktok OR discord OR twitch OR myspace OR kuaishou OR qzone OR "Seina Weibo" OR qq OR threads OR mastadon OR bluesky OR tumblr OR rumble OR quora ) |  |
|  |  | S2 | TITLE-ABS-KEY ( child OR adolescent OR infant OR youth ) |  |
|  |  | S3 | TITLE-ABS-KEY ( "Self-harm" OR "self harm" OR "self-cut*" OR "self-mutilat*" OR "self-injur*" OR suicide OR suicidal ) |  |
|  |  | S4 | S1 AND S2 AND S3 |  |
| Web of Science |  | S1 | screentime OR “screen time” OR smartphone OR “smart phone” OR “social media” OR internet OR gaming OR texting OR television OR “video games” OR messaging OR “smart device” OR phone OR telephone OR computer OR laptop OR tablet OR “Chat room” or online or “social network” OR blog OR sext* OR “instant messag*” or “text messag*” OR cyber* OR forum OR YouTube OR reddit OR twitter OR Snapchat OR Instagram OR Facebook OR telegram OR WeChat OR WhatsApp OR TikTok OR discord OR Twitch OR myspace OR Kuaishou OR Qzone OR “Seina Weibo” or QQ OR Threads OR Mastadon OR bluesky OR Tumblr OR Rumble OR Quora |  |
|  |  | S2 | Child OR adolescent OR infant OR youth |  |
|  |  | S3 | “Self-harm” OR “self harm” OR “self-cut*” OR “self-mutilat*” OR “self-injur*” OR suicide OR suicidal |  |
|  |  | S4 | S1 AND S2 AND S3 |  |
