# Supplementary material for: The impact of screen time and social media on youth self-harm behaviour and suicide: A protocol for a systematic reviews
Source: PLoS One. 2024 Dec 2;19(12):e0314621. doi: 10.1371/journal.pone.0314621 (PMC11611183; doi:10.1371/journal.pone.0314621)
Supplement: Attachment — Submitted filename: Screentime_Response Letter.docx [file pone.0314621.s003.docx]

Professor Emily Chenette

Editor-in-Chief

PLOS ONE

Friday 28^th^ June, 2024

Dear Professor Chenette,

**RE: The impact of screen-time and social media on child self-harm and suicide: a protocol of a systematic review.**

Thank you for taking the time to review our manuscript. Please see out responses to reviewer requests for amendments below. Reviewer comments that did not request alteration have not been included.

| Reviewer comment | Response |
| --- | --- |
| Title: Child implies an age range younger than 12 years old, but a study inclusion criterion is children and adolescents less than 18 years old. Also, I suspect that readers may not be familiar with the NSSI abbreviation. The authors might consider: “The Impact of Screen Time and Social Media on Youth Self-Harm Behavior and Suicide: A Protocol for a Systematic Review” | Thank you for this suggestion. We have amended the title in line with these suggestions. |
| The authors switch from using the phrase “screen time” to the compound word “screentime” in the Background section and throughout the main text. For clarity and consistency, please choose one or the other. (Figure 1: Search terms, is an exception and in Figure 1 authors are correct to use both “screen time” and “screentime.”) | Thank you for highlighting this. We have now changed all instances of the term to be screen time. |
| The first paragraph, first sentence, “The objectives of the study are to investigate the impact of screen time on NSSI, suicidal ideation, suicide attempts and completed suicides in children and adolescents less than 18 years of age”…the preferred phrasing is, “…he/she died by suicide,” and not use “committed suicide” or “completed suicide.” Please reword here and throughout the text (see: [https://www.camh.ca/-/media/files/words-matter-suicide-language-guide.pdf](https://aus01.safelinks.protection.outlook.com/?url=https%3A%2F%2Furldefense.com%2Fv3%2F__https%3A%2F%2Fwww.camh.ca%2F-%2Fmedia%2Ffiles%2Fwords-matter-suicide-language-guide.pdf__%3B!!NVzLfOphnbDXSw!HF2-SA-VsMKR3tvzrnlnYRI7dqIg4yEM75FMh3_qNomWeU-pjVzBBKY_aoYYSXoGtxzuMFw_PSphvFKfWXA2Pl24PcQ%24&data=05%7C02%7Ck2.gillespie%40hdr.qut.edu.au%7C1c229b6e4c7f44eb925f08dc957fdff1%7Cdc0b52a368c544f7881d9383d8850b96%7C0%7C0%7C638549624672332581%7CUnknown%7CTWFpbGZsb3d8eyJWIjoiMC4wLjAwMDAiLCJQIjoiV2luMzIiLCJBTiI6Ik1haWwiLCJXVCI6Mn0%3D%7C0%7C%7C%7C&sdata=oC7LI6dG8JdqvAdGmP5jAEbPRQwOabmUERp2mJUEOHs%3D&reserved=0)). | Thank you for bringing this to my attention. Instances of the phrase committed/completed suicide have been replaced with suicide, death by suicide, died by suicide, or lives lost to suicide, as per the recommended guidelines. |
| Methods and Analysis: Instead of placing definite dates of conducting the study, simply state, “The systematic review is anticipated to be conducted over (whatever) months.” | This has been amended, and dates updated based on current progress, to say:  The systematic review is anticipated to be conducted over December 2023 to August 2024. |
| Eligibility Criteria: The first sentence, “This systematic review will include all peer reviewed publications that describe primary data,” a time interval is typically given, from when-to-when. For example (and the authors may choose whatever time interval they wish), “This systematic review will include all peer reviewed publications that describe primary data from 1972, the introduction of Pong by Atari, to 2023,” or, “This systematic review will include all peer reviewed publications within the past 10 years that describe primary data.” | We have chosen not to restrict the search by date. Line 145 states “no date or location restrictions will be placed on the search”. |
| In Table 1, what is PICOS? It is not explained in the main text nor in Table 1. Perhaps the authors could define PICOS as a footnote in Table 1: Participants, Interventions, Comparators, Outcomes of interest. | We have now included what PICOS stands for in a footnote under the first table. |
| Who will conduct the electronic database search? Will it be a professional librarian or a member(s) of the study team? | The search will be conducted by a member of the research team after consultation with experts and a preliminary, refining search (see lines 1666-169 on page 9). |
| Study Selection: It would be helpful for clarity to add a sentence describing COVIDENCE as an Internet-based application to manage literature and systematic reviews. | This additional description of Covidence has now been included in lines 183-185 of page 10. |
| I did not understand the relevance of reference #33, Nascimento MB, et al. Role of gluteus maximus and medius activation in the lower limb biomechanical control during functional single- leg tasks: A systematic review. Knee. 2023;43: 163-175. Reference #19 in the Nascimento et al. paper refers to the Checklist for Analytical Cross-Sectional Studies from Joanna Briggs Institute…is that what the authors for the present paper mean to refer to? If so, why not simply borrow the reference #19 from the Nascimento et al. paper? | I see how the inclusion of this reference seems out of place. However, we have included that reference for their use of the specific cut-offs that we are including in our review. The recommended JBI reference #19 refers to the checklist, but does not suggest a cut-off for the interpretation of the checklist. |
| In line 208, “…characteristics (age, gender, ethnicity),…” I am pretty sure the authors mean “sex” and not “gender”…correct? | We did intend this to be sex and not gender. This has now been amended. |
| Will types of screen time (smartphone, messaging, social media platforms, gaming, television, or Internet) be abstracted? | Thank you for highlighting this. We have now included “types of screen time” in the list of data to be extracted from papers (line 212 on page 11). |
| References: Many entries are incomplete. Further, careful attention needs to be given to proper formatting and style, especially what to capitalize, what not to capitalize, and page numbers. Some examples follow: | Thank you for highlighting these errors. We have now re-edited the reference section and amended any errors identified. |
| Reference #1: Curtin MA. State suicide rates among adolescents and young adults aged 10–24: United States, 2000–2018. Journal Issue. Hyattsville, MD; 2020. Contract No.: 11. | As above |
| Should be: Curtin MA. State suicide rates among adolescents and young adults aged 10–24: United States, 2000–2018. Nat Vital Stat Rep. 2020;69: 1-10. | As above |
| Reference #5: Geoffroy M-C, Bouchard S, Per M, Khoury B, Chartrand E, Renaud J, et al. Prevalence of Suicidal Ideation and Behaviors in Children Aged 12 Years and Under: A Systematic Review and Meta-Analysis. SSRN Electronic Journal. 2022. | As above |
| Should be: Geoffroy M-C, Bouchard S, Per M, Khoury B, Chartrand E, Renaud J, et al. Prevalence of suicidal ideation and behaviors in children aged 12 years and under: A systematic review and meta-analysis. Lancet Psychiatry. 2022;9: 703-714. | As above |
| Reference #27: Marciano L, Ostroumova M, Schulz PJ, Camerini AL. Digital Media Use and Adolescents' Mental Health During the Covid-19 Pandemic: A Systematic Review and Meta-Analysis. Front Public Health. 2021;9793868. | As above |
| Should be (note the need for an appropriately placed colon after the number 9): Marciano L, Ostroumova M, Schulz PJ, Camerini AL. Digital media use and adolescents' mental health during the COVID-19 pandemic: A systematic review and meta-analysis. Front Public Health. 2021;9: 793868. | As above |
| Additional changes | Please note that due to changing roles within the research team, and after reviewing work conducted on this project by researchers, I have removed the author Xin Li and replaced with author Matthew Porter. |

Sincerely,

Kerri Gillespie
