# Supplementary material for: The impact of screen time and social media on youth self-harm behaviour and suicide: A protocol for a systematic reviews
Source: PLoS One. 2024 Dec 2;19(12):e0314621. doi: 10.1371/journal.pone.0314621 (PMC11611183; doi:10.1371/journal.pone.0314621)
Supplement: Attachment — Submitted filename: Screentime_Response Letter 20241101.docx [file pone.0314621.s004.docx]

Professor Emily Chenette

Editor-in-Chief

PLOS ONE

Friday, 1^st^ November 2024

Dear Professor Chenette,

**RE: The impact of screen-time and social media on child self-harm and suicide: A protocol of a systematic review.**

Thank you for taking the time to review our manuscript. Please see out responses to reviewer requests for amendments below. Reviewer comments that did not request alteration have not been included.

| Reviewer comment | Response |
| --- | --- |
| On page 6 of the revised manuscript, Methods and Analysis, opening paragraph indicates that the review is anticipated to be conducted over December 2023 to August 2024, but we are already in September 2024…has the review already been accomplished? | Unfortunately, our progress has been delayed due to a series of illnesses, deaths, and births in the families of all of the primary reviewers.  We have updated the timeframes in the manuscript for this, and plan to conduct an updated search of the intervening time period using the same methods outlined, before analysis is conducted. We now estimate that the review will be completed by February 2025. |
| The Reference section is better, but still needs some work. Please make sure the references are properly formatted. | Thank you for highlighting this. We have now formatted the reference section. |
| Introduction: Consider differentiating between active screen time (e.g., social media, gaming) and passive screen time (e.g., TV watching), as evidence suggests they may have different effects on mental health. | We have now included this in the section detailing subgroup analysis. This will be a very important aspect that we will investigate. |
| Children and adolescents are in critical stages of brain development, and prolonged screen time may affect neural pathways related to attention and emotional regulation. Discuss how screen time could impact this. Also, consider potential long-term mental health effects. | Additional text has been included in the background (page 4) that discusses potential mechanisms and impacts on later life mental health. |
| Include a discussion on how screen time, particularly from social media or gaming, triggers dopamine release, leading to addictive behaviors that may contribute to mental health problems like anxiety, depression, or NSSI. | The addictive nature of internet use (including gaming and social media), and the neurobiological similarities to substance use, has been described in more detail on page 4. |
| Methods: The need for subgroup analysis is mentioned, but no criteria is provided. Specify which subgroups will be analyzed (e.g., by age, type of screen time).  Explain how studies with varying definitions of screen time (e.g., self-reported vs. objectively measured) will be standardized and integrated into the analysis. | Thank you for highlighting this. We have included further information regarding the subgroups we will analyse. We have also described how different data collection methods will be dealt with on page 13:  *These will include comparing age groups, sex, screen time use (active versus passive use); type (video or messaging apps, chat rooms, websites, gaming); modality (smartphone, computer, television). A comparison of screen time behaviours before and after COVID-19 will also be conducted and discussed. We anticipate that data will be collected using a mixture of self-report, parent-report, and computer recorded methods. Only data using comparable collection tools and methods will be included in meta-analyses. Random-effects meta-analyses will be used to account for minor heterogeneity.* |
| The manuscript mentions contacting authors for missing data but does not provide further steps if no response is obtained. Consider adding alternative strategies such as data imputation or sensitivity analyses. | While we recognise that failing to deal with missing data using statistical methods such as imputation may lead to bias in the results, it is also understood that these statistical methods can significantly alter and potentially bias the outcomes of a meta-analysis (Kahale et al., 2020).  In order to maintain transparency and replicability in a systematic review that may include studies using a range of analytic methods, the study will use a complete case analysis. However, we will discuss the limitations of missing data in the manuscript, and describe how these may potentially contribute to bias in the findings.  This has now been described under ‘Data Synthesis’ on page 12.  Kahale LA, Khamis AM, Diab B, Change Y, Lopes LC. 2020. Potential impact of missing outcome data on treatment effects in systematic reviews: Imputation study. BMJ. 370, m2898. |
| Discussion: Discuss whether any conclusions can be drawn regarding specific thresholds or “safe” amounts of screen time, particularly in relation to mental health outcomes. | The following sentences have been included in the discussion section:  *Current recommendations regarding time spent on screens have been criticised for being unachievable or out of touch with our current screen-dominated culture. The study will therefore also examine time spent using screens in order to identify possible dose-response relationships or ‘safe’ time thresholds in relation to child mental health. Based on our findings, we aim to develop recommendations for screen time use, as well as ‘safe’ amounts of screen time.* |
| 1. The study does not specify any time limitations on the studies to be included, which could result in the inclusion of outdated research. Given that technology and digital behaviors have changed significantly over the past decade, including studies from vastly different time periods might complicate synthesis and lead to misleading conclusions. | Thank you for highlighting this issue. This had been previously discussed with the research team. On further discussion, we have decided to limit the study to papers published in 2007 and later. This is the year that the iPhone was released and widespread use of social media apps began after this time. This will provide a more comparable social and technical environment for included studies. |
| 2. The study acknowledges that the pandemic has affected screen time and mental health behaviors, yet it does not indicate how studies conducted before and during the pandemic will be compared. Screen time behaviors and their psychological effects may have shifted dramatically during COVID-19, necessitating a more explicit plan for handling this potential confounder. | We have now included this in our description of subgroup analyses. We will need to investigate any differences in screen time use, and in prevalence of self-harm and suicide associated with this screen time, before and after the COVID-19 pandemic. |
| 3. While the study rightly aims to examine different types of screen time, such as social media, gaming, and television, the inclusion of vastly different types of media in a single analysis might lead to significant heterogeneity. The outcomes associated with social media use, for example, may be very different from those associated with passive television watching, which could weaken the study's conclusions | We understand that there is a risk with including such a wide range of screen time types. However, the primary purpose of the review is to identify the differences between these types of screen time, and the ways they are used.  A number of researchers have reported benefits of some services, while detrimental impacts of others. It is therefore important we identify the factors that make particular types of screen time beneficial for children as opposed to harmful.  We will also investigate the differences between passive and active forms of use. This has now been included in our more detailed description of subgroup analyses on page 12-13 (as described above). |

Sincerely,

Kerri Gillespie
